# Lactic Acid Bacteria Isolated from Traditional Dry-Cured Fermented Foods with Probiotic Effect: Selection, Mechanisms of Action and Applications

**DOI:** 10.3390/foods14244332

**Published:** 2025-12-16

**Authors:** José M. Martín-Miguélez, Belén Peromingo, Cristina Castaño, Juan J. Córdoba, Josué Delgado, Irene Martín

**Affiliations:** Higiene y Seguridad Alimentaria, Instituto de Investigación de Carne y Productos Cárnicos (IProCar), Facultad de Veterinaria, Universidad de Extremadura, Avda. de las Ciencias s/n, 10003 Cáceres, Spain; jmmm@unex.es (J.M.M.-M.); belenperomingo@unex.es (B.P.); cristinacs@unex.es (C.C.); jdperon@unex.es (J.D.); iremartint@unex.es (I.M.)

**Keywords:** probiotics, lactic acid bacteria, fermented foods, dry-cured foods, plant-based analogues

## Abstract

Traditional dry-cured and fermented foods are part of the diet of many countries all over the world. These products are a source of lactic acid bacteria (LAB). Some of the LAB isolated from these products have a variety of probiotic effects on the consumers, among others, maintaining gastrointestinal homeostasis, enhancing immunity, providing antioxidant effects, preventing vaginal and urinary tract infections, and treating obesity. In addition, LAB has antagonistic properties against human pathogens and foodborne bacteria. This review summarizes methods for isolation, characterization, and selection of LAB with probiotic effects. Besides the effect of the selected probiotic LAB, focusing on gastrointestinal adhesion and colonization, and the described mechanisms of action, emphasizing their potential to advance nutritional innovations, will also be discussed. Furthermore, the advantages of the application of selected probiotic LAB in traditional dry-cured and fermented foods and in plant-based analogues will also be reviewed.

## 1. Introduction

Traditional dry-cured and fermented foods, such as dry-cured fermented sausages and ripened cheeses, are part of the diet of many countries all over the world [1]. These products are a source of lactic acid bacteria (LAB) since these microorganisms are the most important involved in fermentation and in dry-cured processing [2]. LAB are a group of Gram-positive, non-spore-forming, anaerobic or facultatively anaerobic, rod-shaped (*bacilli*) or spherical (*cocci*) bacteria that primarily produce lactic acid as the main product of fermenting soluble carbohydrates [3].

Many LAB isolated from dry-cured fermented foods are considered probiotic. Probiotics are living microorganisms that, when administered in adequate amounts, confer a health benefit to the host [4]. Among the probiotic activities described for LAB are maintaining gastrointestinal homeostasis, enhancing immunity, providing antioxidant effects, preventing vaginal and urinary tract infections, and treating obesity. In addition, LAB has antagonistic properties against human pathogens and foodborne bacteria.

The use of selected LAB with probiotic effect in large-scale production of dry-cured fermented foods requires a previous selection from traditional fermented foods throughout the ripening process, which includes isolation, characterization, and safety evaluation. In addition, the effect of the selected probiotic LAB, focusing on gastrointestinal adhesion and colonization, and their probiotic mechanism actions, should be properly evaluated.

In this review, the methods for isolation, characterization, and selection of LAB with probiotic effects are evaluated. Furthermore, the effect of the selected probiotic LAB, focusing on gastrointestinal adhesion and colonization, and the described mechanism actions, emphasizing their potential to advance nutritional innovations, will also be discussed. In addition, the advantages of the application of selected probiotics LAB in traditional dry-cured and fermented foods and plant-based dry-cured analogues and fermented foods formulated are also reviewed.

For this revision, searches of published data were conducted using Scopus (https://scopus.com (accessed on 28 November 2025), Web of Science (https://webofknowledge.com (accessed on 28 November 2025), Science Direct (https://sciencedirect.com (accessed on 28 November 2025), and PubMed/Medline (https://pubmed.ncbi.nlm.nih.gov/ (accessed on 28 November 2025) with the keywords: probiotics; lactic acid bacteria; fermented foods, dry-cured foods; plant-based analogues. In this review, data published in primary sources—namely, the original publications presenting new evidence in peer-reviewed journals—were given priority. Secondary sources—systematic review articles or meta-analyses derived from primary source literature—were also considered. In any case, articles and book chapters published (in English) from 2001 to 28 November 2025, were analyzed.

## 2. LAB Activities in Dry-Cured Fermented Foods: Possible Candidates as Probiotic Strains

LAB isolated from dry-cured fermented products have shown different activities, some of which may be related to probiotic activity. In this sense, even the activity against pathogenic bacteria first described in LAB has a certain relationship with probiotic activity [5]. Primary antimicrobial activity stems from the production of organic acids, particularly lactic acid, which creates an acidic environment that inhibits pathogenic bacteria, reducing gastrointestinal infections [6]. LAB can also demonstrate antimicrobial activity through the utilization of antimicrobial peptides, bacteriocins, which can act through strategies such as membrane permeabilization, DNA/RNA synthesis interference, and cell wall disruption [7]. Nisin remains the most extensively studied bacteriocin, approved by the Food and Drug Administration (FDA) and the European Food Safety Authority (EFSA) as a food preservative and demonstrating efficacy against both vegetative cells and spores of pathogenic bacteria [8]. The rapid mechanism of action of bacteriocins makes it challenging for pathogenic bacteria to develop resistance, providing a significant advantage over conventional antimicrobials [9].

LAB also produces complementary antimicrobial compounds, including hydrogen peroxide, diacetyl, acetoin, and various low-molecular-weight metabolites [10]. Hydrogen peroxide production is particularly effective in aerobic environments, where it disrupts pathogen membrane structures and oxidizes essential cellular components [11]. Additionally, LAB synthesizes cyclic dipeptides, fatty acids, and reuterin, which collectively contribute to broad-spectrum antimicrobial activity against bacteria, yeasts, and moulds [12].

The protective effects of LAB extend beyond antimicrobial compound production to include sophisticated competitive exclusion mechanisms. LAB strains with strong adhesion capabilities can effectively block pathogen adherence by competing for host cell binding sites and nutrient resources [13]. This competitive exclusion operates through multiple pathways: spatial competition for attachment sites on intestinal epithelium, nutritional competition for limiting nutrients (particularly iron, vitamins, and carbohydrates), and the production of biosurfactants that modify surface properties [14].

Auto-aggregation and co-aggregation abilities of LAB play crucial roles in probiotics. Auto-aggregation allows beneficial bacteria of the same species to form protective colonies, whilst co-aggregation enables different beneficial species to cluster together, creating microenvironments that exclude pathogens [13]. Previous research demonstrates that probiotic LAB strains can greatly reduce pathogen adhesion rates in different products [15,16].

The formation of biofilms by LAB provides an additional protective mechanism, creating structured communities that resist pathogen colonization and environmental stresses [17]. These beneficial biofilms can persist longer in the intestinal environment and serve as protective barriers on food processing surfaces, contributing to both human health and food safety applications [18].

The integration of LAB in biopreservation strategies represents a paradigm shift towards “clean label” food products. LAB-based preservation systems reduce reliance on synthetic preservatives whilst maintaining food safety and extending shelf life through natural mechanisms [19]. This approach aligns with consumer demands for minimally processed foods with enhanced nutritional profiles [20].

Fermented foods represent complex microbial ecosystems where LAB naturally predominate due to their metabolic advantages and environmental adaptations [21]. These foods serve as both delivery vehicles for live probiotic organisms and sources of bioactive metabolites produced during fermentation [20].

Fermented dairy products constitute the category most extensively studied for probiotic LAB applications, but also other foods such as dry-cured fermented meat products, such as dry-fermented sausages [22]. Yoghurt, kefir, fermented milk beverages, ripened cheeses, and dry-cured fermented sausages harbour diverse LAB populations including *Lactobacillus* species, *Streptococcus thermophilus*, and *Lactococcus lactis* [23]. The controlled fermentation environment and nutritional richness of milk products provide optimal conditions for probiotic survival and metabolic activity [24].

Plant-based fermented foods offer significant advantages for probiotic delivery, particularly for lactose-intolerant individuals and those following plant-based diets [25]. Fermented vegetables such as sauerkraut, kimchi, and pickled products contain diverse LAB populations, including *Lactiplantibacillus plantarum*, *Weissella* species, *Pediococcus* species, and *Leuconostoc* species [1]. These products provide additional benefits through enhanced antioxidant activity and polyphenol content, creating synergistic health effects between plant bioactives and probiotic metabolites [26,27].

Fermented dry-cured meat products represent an emerging frontier for probiotic LAB applications, despite the challenging environmental conditions of salt concentration, low pH, and reduced water activity (a_w_) [28]. Thus, it is important to identify and select strains with probiotic potential and exceptional stress tolerance. Recent studies demonstrate successful incorporation of probiotic strains such as *Lactobacillus acidophilus* into salami-type products, maintaining viable counts >10^8^ CFU/g throughout fermentation and ripening processes [29,30]. These meat-adapted probiotic strains exhibit enhanced pathogen inhibition, improved product safety, and maintained sensory quality.

Cereal-based fermented products, such as sourdough and traditional grain beverages, contain diverse LAB communities that provide both technological and probiotic benefits [31,32]. Recent studies have evaluated some fermented beverages as potential LAB probiotic vehicles, enabling the possibility of developing starter cultures for industrial purposes [33]. Moreover, the complex carbohydrate matrix of cereal-based products provides sustained prebiotic support for probiotic activity.

The transition from traditional fermented foods to commercial probiotic applications requires careful strain selection and an evaluation of the hazards and benefits of the selected strains [34]. LAB strains isolated from fermented foods must undergo rigorous safety evaluation, including assessment of antibiotic resistance, virulence factors, and metabolic byproducts [35,36]. The strain-specific nature of probiotic effects necessitates comprehensive characterization and clinical validation for targeted health applications [37].

The integration of traditional fermented foods into modern diets represents a convergence of ancient wisdom and contemporary health science, offering consumers accessible, culturally relevant pathways to improved health through natural probiotic delivery systems.

## 3. The Benefit of Probiotic LAB in Fermented Foods

**Figure 1:**
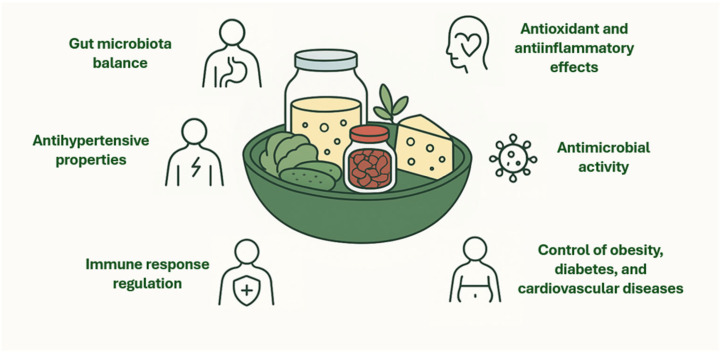
Health-promoting effects of fermented foods.

Fermented foods are known to offer a wide range of health benefits. Modulating gut microbiota, regulating the immune response, and regulating metabolic processes, as well as food preservation, thanks to the biosynthesis of key bioactive compounds, are some of its advantages [20]. Some of the benefits provided by LAB from fermented foods are shown in Figure 1.

Balancing gut microbiota by promoting the growth of beneficial bacteria and inhibiting the proliferation of harmful pathogens is one of the main benefits of consuming probiotic LAB. LAB helps reduce complex substances and eliminate or reduce phytates, tannins, and oxalates [38], improving digestion. Lactic acid fermentation increases the absorption of calcium, iron, zinc, and magnesium by neutralizing the impact of phytic acid [39,40] and helps break down proteins into easy-to-digest peptides and amino acids. On the other hand, some probiotic strains, particularly *Lactobacillus* and *Bifidobacterium*, produce lactase, helping to reduce lactose malabsorption and its associated symptoms [41]. Some LAB can also produce vitamins such as B2, B12, folate, and vitamin K2, thus making foods more nutrient-dense [42]. Finally, this microbiota helps prevent intestinal dysbiosis, which is linked to numerous health problems, such as inflammatory bowel diseases and metabolic disorders [43,44].

LABs demonstrate immunomodulation, as they help activate innate and adaptive immune responses by promoting the proliferation and maturation of immune cells, including lymphocytes (T and B cells), and inducing natural killer (NK) cell activity. Furthermore, they enhance macrophage phagocytosis, which is essential for engulfing and destroying pathogens such as *Listeria monocytogenes*, *Staphylococcus aureus*, *Escherichia coli* O157:H7, and *Salmonella enterica* subsp. *enterica* [45,46,47], etc. Boost antibody production; *Bifidobacterium bifidum* and *Lactobacillus fermentum* strains significantly improve intestinal IgA levels, although in an individual-specific manner, indicating that the effects of probiotics on IgA production may vary by strain and the unique composition of the host’s gut microbiota. On the other hand, studies have shown that probiotic yoghurt can help control allergies and inflammatory diseases by reducing systemic inflammation and modulating the immune response [48]. This immune system enhancement has been correlated with the bioactive compounds generated by probiotics in fermented foods, such as extracellular polysaccharides, bacteriocins, and butyric acid [49,50].

Another beneficial effect of probiotic LAB in fermented foods is their antioxidant and anti-inflammatory effects. Some examples of these foods are kimchi and sauerkraut, fermented milk and soy, and dry-cured fermented meat products [51,52]. This activity is primarily due to changes in compounds such as phenolics, peptides, and flavonoids during the fermentation process. They scavenge free radicals and alleviate oxidative stress, which directly influence ageing, inflammation, and disease development [53,54,55]. On the other hand, antitumor activities have been validated with bioactive peptides that exhibit activity against HT1080 fibrosarcoma cells [56], lung cancer cells, or prostate PC-3 cells. Several bioactive peptides with antihypertensive properties are produced from milk proteins, thus contributing to cardiovascular health [57,58].

On the other hand, consumption of probiotic LAB can promote metabolic balance, which is important for the control of obesity, diabetes, and cardiovascular disease. Its effect is due to the production of short-chain fatty acids (SCFAs) such as acetate, propionate, and butyrate, which have demonstrated beneficial effects, such as improving intestinal membrane integrity, aiding in mineral absorption, reducing blood glucose levels and body weight, stimulating immunity, and regulating several biomarkers related to metabolism, cardiovascular health, and inflammation [59].

Probiotic LAB strains also have antimicrobial activity through competitive inhibition and the production of antimicrobial substances such as bacteriocins and organic acids, as has been previously discussed in the former section.

This provides compelling evidence that the presence of probiotic LAB in dry-cured fermented foods holds great promise for improving functional attributes related to health.

## 4. Compatibility of Probiotic LAB with Fermented Foods Matrices

Fermented dairy, cereal, vegetable, meat and meat-based products serve as natural habitats for LAB and as effective vehicles for delivering viable probiotic cells to consumers. However, probiotic survival and functionality strongly depend on compatibility with the physicochemical and microbial conditions of the host matrix.

Compatibility refers to the capacity of a LAB strain to remain viable and functionally stable without compromising sensory, nutritional, or safety attributes [60]. In fermented foods, parameters such as pH, buffering capacity, a_w_, redox potential, and nutrient composition critically influence probiotic performance.

Although LAB tolerate moderate acidity, matrices with low buffering capacity—such as many plant-based beverages—may undergo rapid pH decreases that reduce viability, whereas dairy matrices provide stronger buffering through caseins and phosphate ions, favouring persistence and activity [60,61]. In addition, a_w_ and osmotic balance affect membrane integrity and metabolic function; low a_w_ or high salt/sugar levels impose osmotic stress, though strains such as *Lacticaseibacillus rhamnosus* and *Lp. plantarum* adapts by accumulating compatible solutes and synthesizing stress-response proteins [62]. Because of such interactions, matrix effects remain highly strain-dependent, and clinical efficacy cannot be inferred from strain data alone.

Matrix composition can either support or limit probiotic performance. Proteins act as buffers and membrane stabilizers, lipids mitigate oxidative stress, and carbohydrates supply fermentable substrates. Prebiotic fibres such as inulin and fructo-oligosaccharides (FOS) enhance LAB adhesion and growth, generating a symbiotic effect. Conversely, plant substrates may contain inhibitory polyphenols or phytates, although some LAB can convert them into smaller antioxidant phenolics [63].

Technological parameters (temperature, duration, inoculation sequence, and oxygen exposure) also influence LAB–matrix compatibility. Mesophilic LAB grow optimally at 30–37 °C, while thermophilic species prefer higher temperatures; excessive acidification or prolonged fermentation decreases viability [64]. Oxygen induces oxidative stress, which can be reduced through co-culturing with yeasts or adding antioxidants such as ascorbate [61,65]. Sequential inoculation, introducing probiotics after primary fermentation, can further enhance their survival [60]. In protein-rich matrices, such as dry-cured fermented meat products, gradual acidification and reduced a_w_ drive microbial succession; selected LAB starters stabilize microbial communities, inhibit spoilage organisms, and maintain viability [66,67,68]. Evidence from in vitro, animal, and human studies shows that probiotic strains delivered through dry-cured fermented sausages can remain viable through gastrointestinal transit and exert measurable functional effects [28].

Several technological strategies enhance compatibility. Selecting stress-tolerant strains remains essential [42]. Microencapsulation with alginate, carrageenan, or starch protects LAB from acidic and oxidative stress while enabling controlled release in the gastrointestinal tract [69]. Symbiotic formulations help sustain viability during storage, and in plant matrices, protein or antioxidant enrichment mitigates low buffering capacity and oxidative instability [63]. Maintaining viable counts above 10^6^–10^8^ CFU g^−1^ throughout shelf life remains the benchmark for functional efficacy [70].

Enhancing compatibility between probiotic LAB and fermented food matrices requires integrating microbial physiology with process design. Advances in omics tools are clarifying stress-adaptation mechanisms and enabling targeted strain–matrix optimization [71]. As demand for plant-based and sustainable foods increases, balancing probiotic stability with sensory and nutritional quality becomes crucial. Ultimately, reinforcing LAB–matrix compatibility will enable the production of stable, functional, and evidence-based probiotic foods [48].

## 5. Selection of LAB with Probiotic Effect from Traditional Dry-Cured Fermented Foods

**Figure 2:**
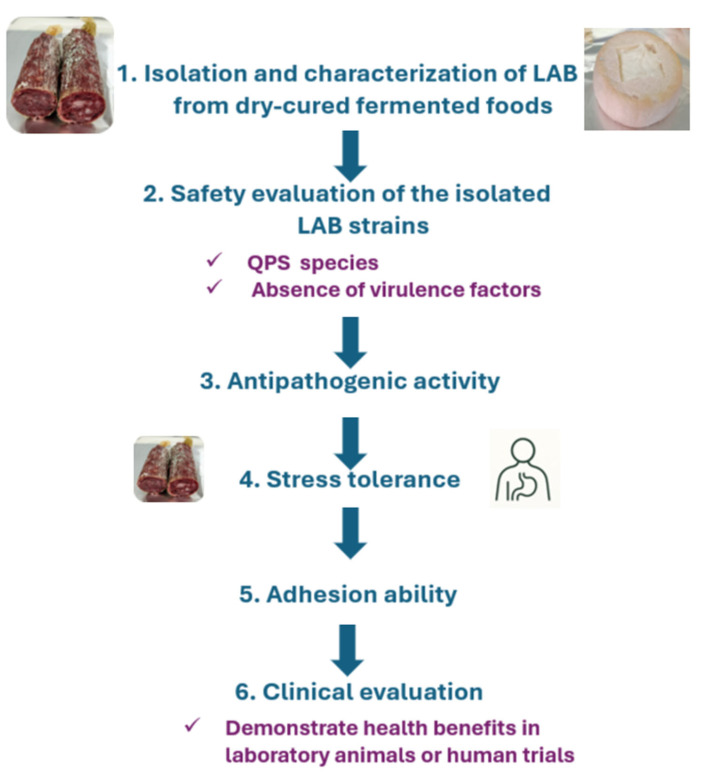
Steps for the selection of LAB strains with probiotic effect from dry-cured fermented foods.

The selection of LAB as probiotics from traditional dry-cured fermented foods requires following a series of steps, beginning with isolation and characterization from these products. These steps, shown in Figure 2 and described below, should enable the selection of good LAB candidates as probiotic microorganisms, eliminating strains that do not pass the selection stages [72,73].

### 5.1. Isolation and Characterization of LAB from Dry-Cured Fermented Foods

The selection process for LAB with probiotic effects from traditional dry-cured fermented foods requires first the isolation of candidate strains from these foods. Thus, samples of dry-cured fermented foods should be taken from the final steps of the ripening process or from the finished product to ensure that they can withstand the conditions of the fermentation, drying, and maturing process. Isolation must be carried out in suitable media such as Man Rogosa Sharpe (MRS) [74]. This methodology has been improved to isolate psychotropic LAB strains by using m-MRS medium and culture conditions developed via statistical optimization using response surface methodology (RSM) [75]. In addition, culture medium with bovine milk or fermented milk supplemented with essential nutrients such as peptides, amino acids, and yeast extracts could be used for isolation and selection of LAB with probiotic effect [76]. Then, these strains should be characterized to establish the genus, species, and subspecies. Since some probiotic activities could be strain-specific, appropriate identification is necessary [73]. Characterization could be made by using biochemical tests based on carbohydrate fermentation, such as API 50 CHL [77]. In addition, molecular methods, such as the 16S ribosomal DNA sequencing, could be used for LAB identification, although this method is appropriate if the nucleotide sequence information of the targeted bacteria is known beforehand [78]. Pulse field gel electrophoresis (PFGE) that allows the separation of the large DNA fragments from restriction digest, PCR, or real-time PCR, with specific primers and probes are also available for the identification of LAB strains [79]. Recent advances in whole-genome sequencing have facilitated the characterization of LAB genomes [80]. This author demonstrated the high sensitivity of whole genome sequencing and Vitek MS (MALDI-TOF) to identify the LAB at the species level. In addition, the combination of these two methods allows the detection of genes encoding probiotic properties of LAB and certain toxic metatolite-related genes and antibiotic resistance-related genes, which are crucial for the possible industrial application of selected LAB [80].

### 5.2. Safety Evaluation of the Isolated Strains

Strains isolated and characterized as LAB should be within the LAB species considered as QPS by the European Union Novel Food regulation, or PROSAF by the United States (FDA) and WHO, or NHPR for Health Canada [72]. The isolated strains should be evaluated for enterotoxin production, hemolytic activity, bile salt deconjugation, and virulence factors. Some of the virulence factors could be evaluated by screening tests of the production of the negative compounds or enzymes: amines [81], hydrolytic enzymes [82,83], and hemolysin [84]. Some of the virulence factors, such as the presence of the following virulence genes, could be evaluated by PCR (Table 1) according to methods previously described [84,85,86]: surface adhesin genes (*esp*, *ace*, *efaA*), extracellular metaloendopeptidase gene (*gelE*), cytolytic activity (*cylA*), hyaluronidase gene (*hly*), aggregation substance precursor (*asa1*), and genes related to the production of biogenic amines (*hdc1*, *tdc* and *odc*). In addition, resistance to antibiotics should be evaluated by test of susceptibility to the following antibiotics: ampicillin, chloramphenicol, erythromycin, tetracycline, gentamicin, streptomycin, and kanamycin, or by PCR evaluating genes of resistance to antibiotics (Table 1) such as aminoglycosides (*aphA-1* gene), β-Lactams (*blaIMP* gene), macrolides (*ermA/TR* gene), quinolones (*gyrA* gene), streptomycin (*rpsL* gene), tetracyclines (*tetA* gene), and vancomycin (*vanA* and *vanB* genes) [84].

The candidate LAB strains with probiotic effects must be negative for the above virulence factors and antibiotic resistance production or PCR.

### 5.3. Antipathogenic Activity

The isolated and characterized LAB strains QPS, such as *L. sakei* or *L. casei* that pass the safety assessment, must be evaluated for their effect on pathogenic bacteria such as *L. monocytogenes*, *Salmonella*, or *Campylobacter jejunii*. Many LAB strains produce extracellular antimicrobial compounds, previously discussed in Section 2 of this review, that they can release when growing in foods [84,90,91] to which they have been added as probiotics, or once adhered to the gut [72].

### 5.4. Stress Tolerance

The selected LAB strains must reach the human gut at high levels to have probiotic effects. Dry-cured fermented foods marketed with health claims due to the addition of probiotics must contain viable cells from probiotic cultures of at least 6–7 log CFU/g in the portion to be consumed [92]. Thus, first, these strains should be capable of surviving the stressful conditions, such as acid, osmotic, and oxidative stresses that may occur during fermentation/maturation in the foods to which they will be added. LAB develops self-regulatory mechanisms to counteract the negative effects of harsh conditions and to survive [93,94]. LAB strains must be isolated from the finished dry-cured fermented foods or at least from the final stages of ripening, since these strains are more adapted to the acid, osmotic, and oxidative stresses. Probably, LAB strains selected at the ripening/fermentation process have been protected from damage by inducing some of the main stress-responsive pathways of LAB, including carbohydrate, amino acid, or energy metabolism responses, DNA repair response, and cell wall/cell membrane regulation, all of them in response to harsh conditions [93]. In addition, the candidate LAB strains should be able to tolerate the stress conditions of the human digestive tract: at time of administration being resistant to the lysozyme present in the oral cavity [95], to the antimicrobial factors of the stomach such as low pH, gastric juice, and pepsin and finally to the bile salts and pancreatin in the intestines, activating some of the above-described stress-responsive pathways of LAB [93]. Some probiotic candidate strains cannot survive in high levels of the low pH gastric juice, which limits their effectiveness in functional foods [96]. Besides stress tolerance, the candidate LAB strains must resist the mild heat shock caused by the internal human body temperature [72].

All the above factors should be evaluated by cultivating the candidate strains at different pH levels with the presence of lysozyme, pepsin, gastric juice, pancreatic juice, and taurodeoxycholic acid [97]. In addition, the viability of candidate LAB strains could be evaluated through in vitro gastrointestinal simulation of the digestion [98]. Some authors found a reduction in the concentration of probiotic microorganisms after simulation of gastrointestinal digestion for most of the commercial probiotics tested, and only a few showed a concentration in the ileum of the small intestine above 6 log CFU/g that could be estimated as the ideal to promote the probiotics’ effect. Knowledge of the stress tolerance mechanism of LAB strains selected for use as probiotics is useful for developing strategies that enable them to survive and thus exert their probiotic action [94]. Thus, the stress resistance of selected LAB with probiotic effect can be further improved by adding exogenous protectants such as galactose, arginine, glutamic acid, glycerol, oleic acid, alginate, calcium or natural antioxidants [93], since these compounds active the carbohydrate, amino acids metabolism response, DNA protection and repair response, and cell wall/cell membrane regulation, in response to stresses conditions as has been discussed in Section 3 of this review.

On the other hand, in the selection of LAB with probiotic effects, screening for genes involved in stress adaptation in LAB strain candidates to probiotics could be very useful for an appropriate selection of strains with probiotic properties. The main genes involved in stress adaptation of LAB are *groEL* and *clpP* are classical stress response genes, widely recognized for their functions in protein folding and proteolysis under adverse conditions of low pH and bile salt resistance [99]. The *bsh* (bile salt hydrolase) gene encodes an enzyme responsible for the hydrolysis of bile salts, a process essential for bacterial survival during gastrointestinal transit [94]. These authors also reported that LBA 1272 and LBA 1446 are implicated in resistance to bile salts. Thus, expression of these genes improves the ability of LAB to persist under the harsh conditions of the gastrointestinal tract. The screening for the above genes involved in stress adaptation by PCR using primers designed based on these genes could be very useful for the selection of probiotic LAB strains (Table 2). Those LAB strains positive for all or some of the PCRs shown in Table 2 will be selected for the next step.

### 5.5. Adhesion Ability

The next step in selecting LAB with probiotic effects is to evaluate the ability of candidate strains to colonize intestinal epithelial cells, which appear to be influenced by the extracellular components of these bacteria and the surrounding composition [100]. The adhesion of the LAB to epithelial cells depends on both the auto-aggregation ability and the hydrophobic properties of the cell surface [72]. The auto-aggregation can be determined by the absorbance of a strain suspension with phosphate-buffered saline [72]. A more precise method to determine the adhesion is to evaluate the ability of candidate LAB strains to adhere to epithelial cells, Caco-2, HT-29, or fetal 1-407 (Figure 2) [101].

### 5.6. Clinical Evaluation

Finally, the candidate LAB strains should demonstrate their health benefits, such as anti-cancer, anti-cholesterol, anti-obesity, anti-diabetic or immunostimulatory activities, or functional molecule secretion, in at least one trial: in laboratory animals or humans to qualify for probiotic status, preferably followed by a confirmatory trial [72,102]. Human trials must be conducted according to generally accepted scientific standards. On rare occasions, the term “probiotic” may be appropriately used to refer to strains of a species (or other taxonomic group) when it has been demonstrated that several members of that species provide a benefit driven by a shared mechanism [73,103].

## 6. LAB with Probiotic Effect Isolated from Fermented Foods

The isolation and selection of LAB with probiotic potential from fermented foods has received increasing attention in recent years, as these products represent natural reservoirs of beneficial microorganisms. Traditional fermented foods such as yoghurt, kefir, artisanal cheeses, and dry-cured fermented meat products (e.g., salami and dry-fermented sausages) harbour a broad diversity of LAB species that have evolved to thrive in complex microbial ecosystems and are frequently linked to health-promoting properties [71,104,105].

Recent advances in microbial genomics, functional screening, and high-throughput characterization techniques have accelerated the identification of LAB with desirable probiotic attributes [106,107].

Beyond dairy, fermented foods of both plant and animal origin continue to emerge as valuable sources of novel LAB strains with probiotic effects. Studies have highlighted the isolation of probiotic candidates from vegetables, cereals, cocoa, coffee, and dry-cured fermented meat products, many of which demonstrate robust survival under gastrointestinal conditions and production of bioactive metabolites. For example, cocoa and coffee fermentations have been shown to harbour *Lactiplantibacillus*, *Limosilactobacillus*, and *Lactococcus* species with probiotic traits comparable to commercial strains [107]. These findings underscore the importance of exploring diverse ecological niches for probiotic discovery.

Current selection strategies increasingly combine traditional microbiological methods with genomic, proteomic, and metabolomic approaches, providing a more comprehensive evaluation of strain safety, technological suitability, and functional capacity [105]. This integrative perspective strengthens the role of fermented foods as sustainable sources of next-generation probiotics with applications in functional foods, nutraceuticals, and biotherapeutics.

Among the LAB most frequently studied for their probiotic potential, *Lp. plantarum*, *Lc. rhamnosus*, and *Levilactobacillus brevis* stand out due to their resilience and multifunctionality. These species not only contribute to fermentation but also produce metabolites of clinical relevance, such as bacteriocins, antioxidant compounds, and exopolysaccharides, which support gut homeostasis, immune regulation, and host metabolic health (Table 3) [105,108,109].

## 7. Probiotics in Plant-Based Analogues

The growing market for plant-based analogues presents an exceptional opportunity to integrate probiotic LAB as functional ingredients, leveraging both their health-promoting properties and their technological capabilities to enhance product quality, safety, and nutritional value. While the application of probiotics in traditional fermented foods has been extensively documented throughout this review, their use in plant-based analogues represents an emerging frontier that warrants focused attention and rigorous investigation.

The inherent suitability of plant proteins as probiotic carriers further validates this application, offering a promising vehicle for delivering health benefits to consumers who choose plant-based diets [111]. However, the application of probiotics specifically in plant-based meat analogues remains relatively limited compared to dairy alternatives, indicating substantial opportunities for innovation and research in this sector [112].

As starters or protective cultures, LAB have demonstrated the capacity to improve multiple quality parameters in plant-based fermented products. Fermentation with LAB increases the bioavailability of nutrients, reduces antinutritional factors such as phytates and tannins, and facilitates the hydrolysis of allergenic proteins, thereby reducing their immunoreactivity [113]. These modifications substantially enhance the nutritional profile of plant-based analogues while simultaneously improving their digestibility and sensory quality (Table 4). The enzymatic activities of LAB can modulate texture through modifications to fibre and protein structures, creating more desirable mouthfeel and consistency in final products [114]. Nevertheless, a significant technological challenge persists in achieving flavour profiles that mimic traditional animal-derived products, as LAB must produce not only organic acids but also the complex array of volatile compounds responsible for characteristic meat or dairy flavours.

As protective cultures, LAB offer biopreservation capabilities that enhance both the safety and shelf life of plant-based analogues, showing control of pathogens and spoilage microorganisms in these products [120]. Extensive research in traditional meat products has documented the antimicrobial efficacy of LAB strains against major foodborne pathogens, including *L. monocytogenes*, *Salmonella* species, and *S. aureus* [121]. The application of such biocontrol strategies to plant-based analogues could address emerging food safety concerns associated with novel plant protein ingredients and processing methods. Indeed, specific research is being performed to evaluate LAB as protective cultures in fermented plant-based analogues [122]. However, the effectiveness of protective cultures must be evaluated within each specific plant-based matrix, as antimicrobial activity and sensory impacts can vary depending on the substrate composition, pH, a_w_, and environmental parameters.

A critical question regarding the application of probiotics in plant-based analogues concerns the optimal source of LAB strains, whether isolates should be derived from plant materials, from the animal products they aim to replace, or from human gastrointestinal sources. The selection of probiotic strains’ origin should ultimately be guided by functional considerations rather than source bias. Strains whose fermentation characteristics closely resemble those of the natural microbiota of the substrate are less likely to introduce undesirable sensory modifications, facilitating consumer acceptance [123]. Researchers have evaluated the use of animal-derived probiotics in plant-based products with satisfactory results [124]. Consequently, a pragmatic approach would involve screening diverse LAB collections from multiple sources (plants, fermented foods, and human origin) to identify strains that optimally balance technological performance, probiotic functionality, sensory acceptability, and safety [63,125].

Considerable progress has been made in applying probiotics to plant-based dairy alternatives, though investigation of probiotics in plant-based meat analogues remains limited. The unique composition and processing conditions of meat analogues, as specific protein sources (pea, soy, wheat gluten), texturization methods (extrusion, shear-cell technology), and lipid profiles, require tailored approaches to probiotic selection and application.

## 8. Multi-Omics Strategies to Probiotic Selection and Applications

The selection of probiotic strains has historically relied on a series of phenotypic screenings [126]. While effective, these methods offer limited insight into the underlying genetic and molecular mechanisms responsible for the observed probiotic traits. The advent of high-throughput sequencing and advanced analytical technologies has been crucial in the era of “multi-omics,” providing a systems-biology framework to dissect probiotic functionality with unprecedented resolution [127]. This integrated approach combines genomics, transcriptomics, proteomics, and metabolomics [128]. Multi-omics is revolutionizing the field by enabling a mechanism-driven selection process that moves beyond mere observation to a deep understanding of a strain’s potential for safety, efficacy, and industrial applicability.

Genomics serves as the foundational layer of any multi-omics strategy, providing the complete genetic blueprint of a potential probiotic strain. Whole genome sequencing, reported in Section 5, is now a routine and cost-effective tool for the initial characterization and safety assessment of novel LAB isolates from fermented foods [129]. A primary application is the in silico safety evaluation, screening for virulence factors and transferable antibiotic resistance genes (ARGs) [130,131].

Beyond safety, genomics uncovers the strain’s functional potential. Genomes are mined for genes encoding key probiotic attributes like stress tolerance, adhesion, and the synthesis of bioactive molecules [132]. For instance, the genomic analysis of *Lp. plantarum* CRM56-2 isolated from tea leaves, revealed the presence of a bile salt hydrolase (BSH) gene, whose activity was later confirmed in vitro, demonstrating the strain’s potential to withstand intestinal conditions [133]. This highlights how genomics provides a powerful predictive tool for targeted functional screening.

While genomics reveals what a cell can do, transcriptomics and proteomics reveal what a cell is performing under specific conditions. These functional omics are essential for understanding how probiotics interact with their environment and with other microbes.

Transcriptomics, typically using RNA-seq, provides a dynamic snapshot of gene expression in response to environmental cues like gut stress [69]. Dual RNA-seq is particularly powerful for deciphering the molecular dialogue between probiotics and host cells [134].

Proteomics complements transcriptomics by identifying the final protein effectors. Mass spectrometry-based techniques are now widely used in proteomic studies to analyze the complete proteome, including sub-proteomes like secreted proteins (“secretome”) and cell surface proteins (“surfome”). Understanding the surfome is critical, as it contains the proteins directly responsible for mediating the adhesion of the bacteria to intestinal epithelial cells—a key criterion for successful colonization [135]. The secretome contains molecules that mediate long-distance communication with the host, including enzymes and immunomodulatory proteins that can influence inflammatory responses [136]. Crucially, proteomics is a key tool for studying how probiotics interact with other members of the gut microbiota. For example, proteomic analysis is used to understand how potential probiotics compete for resources, like prebiotics. A study analyzing the competition for inulin revealed significant proteomic changes in *Lacticaseibacillus paracasei* M38, which adapted its protein expression to try and overcome the strong competitive pressure from other gut commensals [137]. In contrast, proteomics can also reveal synergistic interactions. The analysis of co-cultures of *Bacteroides ovatus* and *Bifidobacterium longum* showed clear evidence of sugar cross-feeding through proteomic changes, where one species provides metabolic by-products for the other to consume, a fundamental process for a healthy gut ecosystem [138]. These studies showcase the power of proteomics to move beyond single-strain characterization to understanding community-level interactions.

Metabolomics focuses on the ultimate phenotypic output: the small-molecule metabolites produced by probiotics [139]. These are often the primary mediators of health benefits, such as short-chain fatty acids (SCFAs) derived from Fibre fermentation [140]. The antioxidant capacity of potential probiotics is another desirable metabolic trait, as demonstrated for *Lp. plantarum* CRM56-2, suggesting its ability to produce metabolites that can counteract oxidative stress [133].

The limitations of conventional screening methods, which typically focus on single phenotypic traits like acid tolerance or antimicrobial activity, often restrict the comprehensive understanding of a strain’s functional potential in complex biological environments. In contrast, omics-based approaches provide a powerful, high-resolution alternative for the selection of superior candidates. For example, while standard culture assays identify strains exhibiting resistance to host bile, as mentioned in Section 5, genomic analysis can precisely map the specific efflux pumps or bile salt hydrolase genes responsible for this trait [141], offering deeper mechanistic insight. Furthermore, proteomics is often applied to compare the differential quantities of cell-surface proteins in potential probiotic strains under gut-mimicking conditions, revealing key biomarkers for enhanced adhesion or immunomodulation [142,143] that are missed by simple in vitro adherence assays. This integrated, system-level data allows for a more targeted and evidence-based selection strategy compared to traditional phenotypic screening, accelerating the development of next-generation food-derived probiotics. The main drawbacks of these novel techniques are the requirements of sophisticated and expensive equipment, as well as trained researchers to manage the massive data yielded by these techniques.

Therefore, true power of the multi-omics approach lies in integrating data to build a comprehensive, system-level understanding. A modern selection pipeline begins with genomic screening to identify safe candidates with functional potential. This potential is then validated using proteomics and transcriptomics to understand how the strain behaves and interacts with its environment and other microbes. Finally, metabolomics confirms the production of bioactive compounds.

This integrated approach allows for a rational, evidence-based selection of next-generation probiotics. By combining genomics to predict function with proteomics (e. g. in *Lp. plantarum* CRM56-2) to understand complex microbial interactions (e.g., competition and cross-feeding), researchers can move beyond isolating robust strains to designing microbial consortia with specific, predictable, and synergistic health benefits.

## 9. Industrial Requirements for the Exploitation of LAB Selected as Probiotics

LAB strains selected as probiotics must first be deposited in a standard microorganism culture collection. This is a necessary condition for the possible industrial exploitation of these strains [102].

On the other hand, LAB selected as probiotics and added to dry-cured fermented foods must have been evaluated to ensure that they do not cause sensory changes in the products or reduce their shelf life [72].

LAB preparations with proven probiotic effects could be considered as medical or pharmaceutical products when they offer health benefits. Probiotics are categorized as food in the U.S., European Union (EU), Japan, Thailand, Malaysia, Singapore, and Indonesia, with Malaysia, Singapore, and Indonesia also classifying them as “health supplements [144]. Finally, the LAB incorporated as probiotics into a food must comply with legal regulations. In the European Union (EU), the food operators placing probiotics on the market must comply with the Regulation EC 178/2002 [145] to ensure that their products are safe. It is also necessary that food operators identify and control food safety hazards (Regulation [EC] No 852/2004) [146]. Currently, there are no safety criteria in the regulations, and no guidance is available for assessing the safety of probiotics in food supplements [147]. The independent agency in food safety (EFSA), funded by the EU, established for the microorganisms that could be commercialized the concept of qualified presumption of safety (QPS) to facilitate a harmonized generic pre-assessment to support safety risk assessments. However, LAB with probiotic effects endowed with QPS status does not guarantee market authorization. The QPS list [148] is used as a point of reference when assessing the safety of probiotics in food supplement notifications.

## 10. Perspectives and Future Remarks

Traditional dry-cured and fermented foods represent an untapped source of LAB with potential probiotic properties and technological relevance. The ecological pressures inherent to these matrices favour the selection of strains with enhanced tolerance to stress, acid, and salt—traits that may improve survival and functionality in the gastrointestinal tract.

Future research should emphasize comprehensive genomic and functional analyses to elucidate genes and pathways related to adhesion, immunomodulation, and antimicrobial activity. The integration of multi-omics approaches with well-designed in vitro and in vivo studies will be essential to validate the probiotic potential and safety of these isolates.

Moreover, the application of selected probiotic LAB as starters or adjunct cultures could enhance the microbiological stability, sensory quality, and functional attributes of fermented foods. Finally, advancing regulatory validation and clinical evidence will be crucial to facilitate the transition from traditional isolates to scientifically substantiated probiotic strains with clear benefits for both consumers and the food industry.

## 11. Conclusions

The traditional dry-cured fermented foods are a source of LAB strains, some of which have probiotic effects on the consumers, among others, maintaining gastrointestinal homeostasis, enhancing immunity, providing antioxidant effects, preventing vaginal and urinary tract infections, and treating obesity. In addition, some LAB have antagonistic properties against human pathogens and foodborne bacteria. It is necessary to follow an appropriate protocol for selecting LAB strains with probiotic capacity that includes isolation from fermented ripened foods, evaluation of the safety of candidate strains, their antipathogenic activity, stress tolerance in dry-cured fermented foods and in the human gut, ability to adhere to the gastrointestinal tract, and clinical evaluation. The use of this methodology has made it possible to obtain strains from these foods with proven probiotic properties. The development of omics techniques has enabled the application of different multi-omic strategies for the selection, characterization, and application of LAB with probiotic effects. LABs with probiotic effects that have been selected from dry-cured fermented foods are available for use in these kinds of foods as well as in animal-based food analogues, which enable the production of foods with functional characteristics and the corresponding industrial exploitation.

## Figures and Tables

**Table 1:** Target genes, primer sequences, annealing temperature, and amplicon size for PCR detection of genes encoding virulence factors or antibiotic resistance.

Target Gene	Encoded Protein	Primer Sequence (5′–3′)	Annealing Temperature (°C)	Amplified Size (bp)	References
*gelE*	Gelatinase	F-TATGACAATGCTTTTTGGGAT R-AGATGCACCCGAAATAATATA	47	213	[86]
*cylA*	Cytolisin	F-ACTCGGGGATTGATAGGC GCTGCTAAAGCTGCGCTT	52	688	[86]
*hyl*	Hyaluronidase	F-ACAGAAGAGCTGCAGGAAATG R-GACTGACGTCCAAGTTTCCAA	53	276	[86]
*asa1*	Aggregation substance	F-GCACGCTATTACGAACTATGA R-TAAGAAAGAACATCACCACGA	50	375	[86]
*esp*	Enterococcal surface	F-AGATTTCATCTTTGATTCTTG R-AATTGATTCTTTAGCATCTGG	47	510	[86]
*efaA*	Endocarditis antigen	F-GCCAATTGGGACAGACCCTC R-CGCCTTCTGTTCCTTCTTTGGC	57	688	[86]
*ace*	Adhesion of collagen	F-GAATTGAGCAAAAGTTCAATCG R-GTCTGTCTTTTCACTTGTTTC	48	1008	[86]
*hdc1*	Histidine decarboxylase	F-AGATGGTATTGTTTCTTATG R-AGACCATACACCATAACCTT	46	367	[86]
*odc*	Ornithine decarboxylase	F-GTNTTYAAYGCNGAYAARCANTAYTTYGT R-ATNGARTTNAGTTCRCAYTTYTCNGG	54	1446	[86]
*tdc*	Tyrosine decarboxylase	F-GAYATNATNGGNATNGGNYTNGAYCARG R-CCRTARTCNGGNATAGCRAARTCNGTRTG	55	924	[86]
*tdc2*	Tyrosine decarboxylase	F-AAYTCNTTYGAYTTYGARAARGARG R-ATNGGNGANCCDATCATYTTRTGNCC	50	534	[86]
*ccf*	Sex pheromones	F-GGGAATTGAGTAGTGAAGAAG R-AGCCGCTAAAATCGGTAAAAT	51	543	[87]
*vanA*	Vancomycin resistance	F-TCTGCAATAGAGATAGCCGC R-GGAGTAGCTATCCCAGCATT	52	377	[88]
*vanB*	Vancomycin resistance	F-GCTCCGCAGCCTGCATGGACA R-ACGATGCCGCCATCCTCCTGC	60	529	[88]
*aphA-1*	Aminoglycoside resistance	F-ATGGGCTCGCGATAATGTC R-CTCACCGAGGCAGTTCCAT	56	600	[89]
*blaIMP*	β-Lactams resistance	F-CTACCGCAGCAGAGTCTTTG R-AACCAGTTTTGCCTTACCAT	53	587	[89]
*gyrA*	Quinolones resistance	F-TTCTCCGATTTCCTCATG R-AGAAGGGTACGAATGTGG	49	458	[89]
*ermA/TR*	Macrolides resistance	F-TCAGGAAAAGGACATTTTACC R-ATACTTTTTGTAGTCCTTCTT	46	432	[89]
*rpsL*	Streptomycin resistance	F-GGCCGACAAACAGAACGT R-GTTCACCAACTGGGTGAC	54	501	[89]
*tetA*	Tetracyclines resistance	F-GTAATTCTGAGCACTGTCGC R-CTGCCTGGACAACATTGCTT	54	937	[89]

**Table 2:** Target genes, primer sequences, annealing temperature, and amplicon size for PCR detection of genes encoding stress resistance factors.

Target Gene	Encoded Protein	Primer Sequence (5′–3′)	Annealing Temperature (°C)	Amplified Size (bp)	References
*groEL*	Heat shock protein 60	F-TTCCATGGCkTCAGCrATCA R-GCTAAyCCwGTTGGCATTCG	58	168	[99]
*LBA 1272*	Cyclopropane FA	F-GGCTTACCAATGGCCACCTT R-GATCAAAAAGCCGGTCACGA	57.5	210	[94]
*LBA 1446*	Multidrug resistance	F-GCTGGAGCCACACCGATAAC R-CAACGGGATTATGATTCCCATTAGT	58	275	[94]
*bsh*	Conjugated bile salt acid hydrolase	F-ATTCCWTGGWTWYTGGGACA R-AAAAGCRGCTCTNACAAAWCKAGA	58	384	[94]
*clpL*	ATPase synthase	F-GCTGCCTTyAAAACATCATCTGG R-AATACAATTTTGAArAACGCAGCTT	56	158	[94]

**Table 3:** Representative lactic acid bacteria (LAB) strains isolated from fermented foods, their sources and reported probiotic effects.

LAB Strain	Food Source	Main Probiotic Effects	References
*Lactiplantibacillus plantarum* 299v	Fermented vegetables/cereals	Gut colonization, modulation of microbiota, immunomodulation, cholesterol-lowering	[107,110]
*Lacticaseibacillus rhamnosus* GG	Fermented milk (yoghurt, cheese)	Survival in GIT, pathogen inhibition, immune modulation, clinical validation	[71,107]
*Levilactobacillus brevis* MK05	Fermented meat (sausages)	Antioxidant activity, bile tolerance, antimicrobial activity	[107]
*Lactococcus lactis* subsp. *lactis*	Traditional dairy products	Immune stimulation, antimicrobial effects, technological suitability as a starter culture	[105]
*Limosilactobacillus fermentum* ME-3	Cocoa fermentation	Antioxidant properties, cholesterol-lowering, gut protection	[107]
*Pediococcus acidilactici* VKU2	Traditional cereal-dairy product (Tarkhineh, Iran)	Cholesterol removal, antioxidant activity, survival under acidic conditions	[105]
Leuconostoc mesenteroides	Kimchi, sauerkraut, vegetable fermentations	Exopolysaccharide production, antioxidant activity, gut microbiota modulation	[104]

**Table 4:** Plant-based substrates and lactic acid bacteria (LAB) are used for their fermentation.

Microbial Strain	Plant-Based Substrate	Plant Source	Fermentation Effects	References
*Lactobacillus delbrueckii* subsp. *bulgaricus; Streptococcus thermophilus; Lactiplantibacillus plantarum; Lacticaseibacillus casei; Lactobacillus acidophilus; Bifidobacterium*	Fermented “yogurt-like” analogue	Soybean; rice; hazelnut	Increased antioxidant capacity and enhanced digestive enzyme inhibition through fermentation. Elevated vitamin B6 and B1 concentrations following fermentation	[115,116,117]
Kefir culture	Kefir analogue	Almond; peanut; hazelnut; walnut; cashew	Titratable acid reduction, prebiotic Fibre supporting probiotic viability	[118]
*Lactobacillus acidophilus, Lacticaseibacillus paracasei, and Bifidobacterium*	Cheese analogue	Pea protein isolate	Fermentation did not affect the characteristics of the final product	[119]
*Lactiplantibacillus plantarum; Leuconostoc mesenteroides*	Vegetable fermentations/analogues	Mixed vegetables	High LAB counts, production of organic acids and bioactives, antioxidant enhancement	[54]

## Data Availability

No new data were created or analyzed in this study. Data sharing is not applicable to this article.
